# Neuromuscular electrical stimulation promotes development in mice of mature human muscle from immortalized human myoblasts

**DOI:** 10.1186/s13395-016-0078-6

**Published:** 2016-02-27

**Authors:** Paraskevi Sakellariou, Andrea O’Neill, Amber L. Mueller, Guido Stadler, Woodring E. Wright, Joseph A. Roche, Robert J. Bloch

**Affiliations:** 1 Department of Physiology, University of Maryland School of Medicine, 655 W. Baltimore St., Baltimore, MD 21201 USA; 2 Present address: FAME Laboratory, School of Exercise Science, University of Thessaly, Karies, Trikala, 42100 Greece; 3 Program in Molecular Medicine, University of Maryland, Baltimore, MD 21201 USA; 4 Department of Cell Biology, University of Texas Southwestern Medical Center, Dallas, TX 75390 USA; 5 Present address: Berkeley Lights, Emeryville, CA 94608 USA; 6 Present address: Department of Health Care Sciences, Wayne State University, Detroit, MI 48201 USA

**Keywords:** Stem cell, Xenograft, Differentiation, Desmin, Lamin, Spectrin, Neuromuscular junction, Muscular dystrophy, FSHD

## Abstract

**Background:**

Studies of the pathogenic mechanisms underlying human myopathies and muscular dystrophies often require animal models, but models of some human diseases are not yet available. Methods to promote the engraftment and development of myogenic cells from individuals with such diseases in mice would accelerate such studies and also provide a useful tool for testing therapeutics. Here, we investigate the ability of immortalized human myogenic precursor cells (hMPCs) to form mature human myofibers following implantation into the hindlimbs of non-obese diabetic-*Rag1* ^*null*^ *IL2rγ* ^*null*^ (NOD-Rag)-immunodeficient mice.

**Results:**

We report that hindlimbs of NOD-Rag mice that are X-irradiated, treated with cardiotoxin, and then injected with immortalized control hMPCs or hMPCs from an individual with facioscapulohumeral muscular dystrophy (FSHD) develop mature human myofibers. Furthermore, intermittent neuromuscular electrical stimulation (iNMES) of the peroneal nerve of the engrafted limb enhances the development of mature fibers in the grafts formed by both immortal cell lines. With control cells, iNMES increases the number and size of the human myofibers that form and promotes closer fiber-to-fiber packing. The human myofibers in the graft are innervated, fully differentiated, and minimally contaminated with murine myonuclei.

**Conclusions:**

Our results indicate that control and FSHD human myofibers can form in mice engrafted with hMPCs and that iNMES enhances engraftment and subsequent development of mature human muscle.

## Background

Muscular dystrophies afflict approximately 1 in 5000 individuals worldwide. Developing murine models to study these diseases is essential in understanding the mechanisms of pathogenesis and in testing potential therapies. Animal models for some muscular dystrophies, such as facioscapulohumeral muscular dystrophy (FSHD), are still unavailable, however, and murine models of some muscular dystrophies, whether naturally occurring or genetically engineered, are limited because they do not replicate all the features of the human disease (reviewed in [1–4]). Mice that carry human muscles from individuals with these diseases would serve as more accurate models, reproducing most, if not all, of the morphological, physiological, and genomic features of the muscular dystrophies in man.

Two approaches have been tested to develop grafts of human muscle tissues in mice. In one, the xenografts are created by introducing small bundles of mature human myofibers, obtained at biopsy, and suturing them to muscles in immunodeficient mice [5]. Over time, some of these grafts survive and reform mature muscle tissue that is largely human in origin, although they can also contain significant numbers of murine myonuclei. The myofibers in these grafts can be innervated by motor neurons and supplied by capillaries that are murine in origin, and they contract in response to electrical stimulation. Although the mice carrying these human grafts provide potentially excellent models, they would be difficult to generate in the numbers needed for therapeutic testing.

As an alternative approach, several laboratories have been investigating the ability of human myogenic cells to develop into mature human muscle tissue in immunodeficient or dystrophic mice [6–11], reviewed in [12]. In these studies, the endogenous murine muscle is typically eliminated by freezing or injection of a toxin and, in some cases, is prevented from regenerating by prior irradiation. Human myoblasts are often tagged with luciferase [13, 14], or green fluorescent protein (GFP) [15, 16], to enable them to be tracked by luminometric or fluorescence methods after injection. In some studies, engrafted mice have been exposed to different pharmacological agents, such as Losartan [17], to alter angiotensin II signaling, or a soluble form of the receptor for myostatin, ActRIIB-Fc [18], to promote myogenesis and reduce fibrosis. These treatments have improved the survival of the engrafted human myogenic cells in mice and their differentiation into myofibers, but the muscles they form are small and therefore difficult to study by physiological, morphological, and genomic methods. Moreover, the engrafted muscle tissue may also contain a large number of murine myonuclei, indicating that they are largely hybrid in nature [18–20].

Here, we describe methods to generate xenografts in mice of mature human myofibers from muscle precursor cells, without significant murine contamination. We use immunodeficient non-obese diabetic-*Rag1* ^*null*^ *IL2rγ* ^*null*^ (NOD-Rag) mice, which we first X-irradiate locally to prevent regeneration of murine muscle and then treat with cardiotoxin (CTX) to eliminate the murine *tibialis anterior* (TA) muscle. We then inject an immortalized clonal cell line of human myogenic precursor cells (hMPCs) that express luciferase, enabling us to track the developing graft over time (lox-hTERT hygromycin + cdk4-neo (LHCN) cells [21]). We periodically subject the engrafted leg to intermittent neuromuscular electrical stimulation (iNMES) via the peroneal nerve. Electrical stimulation has long been known to promote muscle differentiation in vitro [22–24] and in vivo [25, 26], and iNMES has been used therapeutically in man to promote the recovery of skeletal muscle from injury [27–30]. We report that iNMES significantly increases the number and size of the human myofibers and improves the morphology of the human skeletal muscle tissue in the grafts. Many of the myofibers in the graft are similar to mature murine myofibers in size, and they are both innervated by motor neurons and fully differentiated. Moreover, they are comprised almost exclusively of human myonuclei, with minimal contamination by murine myonuclei. Initial studies of xenografts prepared with cells from an individual with FSHD suggest that our methods can be also used with dystrophic hMPCs. Thus, our results indicate that xenografting of hMPCs into mice can generate human muscle tissue with minimal contamination with murine myonuclei and that iNMES promotes the formation and development of the grafts.

## Methods

### Animals

Male NOD-Rag immunodeficient mice (strain NOD.Cg-Rag1tm1Mom Il2rgtm1Wjl/SzJ; Jackson Laboratories, Bar Harbor, ME) were used. These NOD-congenic mice harbor the *Rag1* ^*null*^ mutation on chromosome 2 and the *IL2rγ* ^*null*^ mutation on the X-chromosome which results in the absence of T, B, and NK cells. NOD-Rag mice are suitable for muscle xenografting, as they do not reject transplanted myogenic cells, and they tolerate high levels of irradiation [11, 31]. All protocols were approved by the Institutional Animal Care and Use Committee of the University of Maryland, Baltimore.

### Cells

The immortalized hMPCs used in this study are described in Zhu et al. [21]. They were generated by establishing primary hMPCs cultures by explant techniques from the pectoralis major muscle of a 41-year-old male Caucasian heart transplant donor. Cells were immortalized by infection and selection with retroviruses containing expression cassettes for CDK4 and neomycin resistance, or human telomerase reverse transcriptase (hTERT) and hygromycin resistance; the latter two flanked by Lox-P sites. From this immortal population, a clone with robust myotube formation upon exposure to differentiation conditions in vitro was selected. This cell line was initially named LHCN-M2 (for lox-hTERT hygromycin + cdk4-neomycin, myogenic clone #2). Here, we refer to the cell line as “LHCN.” Culture conditions were as published [32]. We also used hMPCs derived from the biceps muscle of an individual with FSHD that were immortalized in an identical fashion. These cells (15Abic) which we refer to as “FSHD” have been described [32].

### X-irradiation

The left hindlimbs of young adult mice (8 weeks old) were subjected to a single, localized dose of X-irradiation, as previously described [33]. This dose has been previously shown to suppress >90 % of satellite cell activation following CTX treatment [34]. Briefly, mice were anesthetized by an intraperitoneal injection of a 2:1 mixture of 80 mg/kg ketamine (Butler Schein Animal Health, Dublin, OH) and 7 mg/kg xylazine (Akom, Decatur, IL) and placed within a lead box. The left hindlimb was extended through a hole in the box and exposed to X-irradiation at a single dose of 25 Gy at ~2.5 Gy/min. Ionizing radiation was delivered with a Pantak-Seifert 250KpV X-ray Irradiator (bipolar series model HF 320, East Haven, CT). The radiation beam was focused onto the lower hindlimb. Ion chamber dosimetry (PTW model 31006, Freiburg, Germany) was performed outside the collimator to ensure delivery of the exact dosage to the hindlimb, as well as inside the collimator (lead shielding), to monitor backscatter of radiation.

### CTX treatment and hMPC transplantation

Mice were anesthetized with 2–2.5 % isoflurane. A solution of 0.3 mg/ml CTX (*Naja mossambica mossambica*; Sigma-Aldrich, St. Louis, MO) was prepared in sterile phosphate-buffered saline (1× PBS, 0.02 % sodium azide) and sterilized by filtration (0.2 μm filter, PALL Corporation; Port Washington, NY). The CTX solution was loaded into a 300-μl tuberculin syringe with a 29-gauge needle (Terumo, Elkton, MD) and injected to a final dose of 2 μg/mg body weight, distributed to three sites along the length of the host TA muscle. CTX injection induces selective degeneration of endogenous myofibers without affecting the blood vessels or nerves [35, 36]. CTX injections were performed 24 h before hMPC transplantation, as pilot studies suggested that injecting cells at later times following CTX treatment resulted in poorer engraftment. Immediately prior to transplantation, hMPCs were released from the substrate by brief digestion with trypsin, diluted in an equal volume of growth media, and subjected to centrifugation at 1 × 10^3^ rpm for 3 min (IEC Centra CL2 centrifuge, Thermo Scientific). Cells were suspended in 50 μl growth media and injected into the TA compartment along the length of the tibia to promote broad distribution of the injected hMPCs. For studies of LHCN cell engraftment, aliquots of cell suspensions containing 5 × 10^5^ (group 1, *n* = 18) or 2 × 10^6^ (group 2, *n* = 25) hMPCs were injected and monitored over time, with no further treatment. An additional experimental group was injected with 2 × 10^6^ hMPCs and subjected to intermittent neuromuscular electrical stimulation (group 3, *n* = 23), as described below. For studies of FSHD cell engraftment, only aliquots of 2 × 10^6^ cells were used, with six mice treated with iNMES and six controls.

### Bioluminescence imaging

Bioluminescence imaging (BLI) was performed with a Xenogen IVIS® 200 system (Perkin Elmer, Hanover, MD) to monitor the survival and development of the engrafted hMPCs non-invasively. Mice were anesthetized with 2–2.5 % isoflurane. A solution of d-luciferin (40 mg/ml) (Caliper Life Sciences, Hopkinton, MA) was prepared in sterile PBS and sterilized by filtration (0.2 μm filter; PALL Corporation). Mice were injected intraperitoneally (IP) with d-luciferin at 150 mg/kg and returned to their cages for 5 min to allow the compound to distribute through the body. Anesthetized mice were placed in the Xenogen IVIS chamber to measure the light emitted as a result of the action of luciferase on d-luciferin. Imaging was performed 15 min after d-luciferin injection, when the luciferase activity reached its peak, based on previously acquired studies of the luciferase signal (60 min sequence, imaging for 1 min at 5 min intervals). In total, 16 mice injected with LHCN cells were used for assessing the intensity of the bioluminescence signal at 2 h (day 0) and at 7 and 28 days after transplantation. To estimate the loss of transplanted hMPCs over longer time periods, five of these 16 mice were scanned on a weekly basis for up to 7 weeks (group 1 = 3 mice, group 2 = 1 mouse, and group 3 = 1 mouse). Regions of interest (ROI) within the injected region were selected, and the light generated by luciferase was quantified as photon flux (photons/second) with Living Image® 4.3.1 software (Perkin Elmer). The bioluminescence image, shown in pseudocolor, is overlaid on a photographic image of the mouse, with intensity represented from blue to red, representing least to most bright.

### Intermittent neuromuscular electrical stimulation

iNMES was initiated on day 5 after initial transplantation. Mice were anesthetized with 2–2.5 % isoflurane. The ankle dorsiflexors of the injected hindlimb were stimulated electrically through the intact skin over the common peroneal nerve, with the electrode placed over the head of the fibula. Monophasic square wave pulses, 0.1 ms in duration, were delivered to the stimulation electrode by an S48 Stimulator (Grass Instruments, Warwick, RI). A stimulation isolation unit (model PSIU6; Grass Instrument) was used to minimize artifact and to ensure that the peak current delivered was no greater than 15 mA. The iNMES training protocol consisted of four sets of ten contractions. Each contraction lasted 500 ms (150 Hz pulse frequency), followed by a 500-ms rest. Rest times of 2 min were allowed between each set of contractions to minimize the effect of fatigue. iNMES training was performed three times/week for 4 weeks. Most samples were analyzed between 4 and 5 weeks after injection of hMPCs.

### Antibodies, immunofluorescence labeling, imaging, and morphometry

Mice under isoflurane anesthesia were euthanized by cervical dislocation. Muscle in the engrafted region was removed, weighed, and embedded in O.C.T. (Tissue Tek; Torrance, CA), snap frozen in a slush of liquid N_2_ and stored at −80 °C. Serial frozen cross sections (10–15 μm thick) were cut and mounted on glass Superfrost microslides (VWR, Radnor, PA).

Immunolabeling was performed on unfixed cross sections from every TA muscle sample to visualize the presence of human proteins in the graft. Mouse monoclonal antibody specific for human β-spectrin (1:50; Leica, Buffalo Grove, IL) was routinely used to label the sarcolemma. In some studies, mouse monoclonal antibody to human lamin A/C (1:200; Leica) were used to label the nuclear lamina. To evaluate the internal organization of the newly formed human myofibers, sections were also stained with a rabbit polyclonal antibody to desmin (1:100; Thermo Scientific).

For immunolabeling with mouse monoclonal antibodies, the “mouse-on-mouse” (MOM) kit from Vector Laboratories (Burlingame, CA) was used to reduce non-specific staining. Briefly, sections were incubated for 1 h with MOM Ig blocking reagent and then for 10 min with MOM diluent solution (1:80 dilution of protein concentrate in PBS). Slides were incubated overnight at 4 °C with primary antibodies diluted in MOM diluent solution, washed three times with PBS, and incubated with Alexa Fluor 488-conjugated goat anti-mouse IgG (H + L) and Alexa Fluor 568-conjugated goat anti-rabbit IgG (H + L) (1:200; Molecular Probes, Life Technologies, Grand Island, NY) for 1–2 h at RT. Sections were washed three times with PBS and mounted in VectaShield containing 4′,6-diamidine-2′-phenylindole dihydrochloride (DAPI; Vector Laboratories). For sections in which monoclonal anti-spectrin and polyclonal rabbit anti-desmin were used, we followed similar procedures but incubated the sections in Alexa Fluor 488-conjugated goat anti-mouse IgG (H + L) and Alexa Fluor 633-conjugated goat anti-rabbit IgG (H + L) (1:200; Molecular Probes).

Samples were viewed with a Zeiss LSM5 DUO confocal microscope, with 40× or 63× objectives (Carl Zeiss, Jena, Germany) and the pinhole set at 1 Airy unit. Images were analyzed with LSM Image software (Carl Zeiss). Myofibers of human origin, identified by labeling of the sarcolemma with antibodies against human β-spectrin (see above), were counted.

Minimum Feret’s diameter, a measure of the size of myofibers, was determined for grafts generated with LHCN cells, as described [37]. Engrafted myofibers were divided into three groups according to size (1–9, 10–19, and ≥20 μm). For each graft, the percentage of myofibers within each group was calculated and compared between groups. Distances from the largest human myofibers in the graft to their nearest neighbors were also measured. Additional morphometric measurements included the number of human lamin A/C-positive nuclei in the grafts, identified by labeling with antibodies to human lamin A/C [6–8, 10, 11, 38–43], the number of murine myonuclei in myofibers of human origin, identified as myonuclei labeled by DAPI but not by antibodies to human lamin A/C, and the number of centrally nucleated human and murine myofibers.

For labeling of the neuromuscular junction in grafts formed by LHCN cells, sections were fixed with 4 % paraformaldehyde, washed three times with TBS and incubated with TBS + 0.25 % Triton X-100 for 10 min. Sections were incubated with antibody dilution buffer (PBS + 5 % goat serum, 1 % BSA, 0.1 % fish skin gelatin, and 0.05 % azide), followed by overnight incubation with mouse monoclonal antibodies to synaptophysin (1:100 DAKO M7315) and human β-spectrin. After washing, samples were incubated for 1–2 h at RT with Alexa Fluor 488-conjugated goat anti-mouse IgG1, for synaptophysin, and Alexa Fluor 568-conjugated goat anti-mouse Ig2B for β-spectrin, as well as Alexa Fluor 633-α-bungarotoxin (all from Molecular Probes). Secondary antibodies and α-bungarotoxin were diluted to 1:500 and 1:200, respectively. Sections were washed three times with PBS and mounted in VectaShield containing DAPI.

### Statistical analysis

Statistical comparison of the number of fibers in the different groups of xenografts used the *t* test. The sizes of the engrafted human myofibers across the experimental groups were analyzed with the chi-square test. The intermyofiber distance between the largest engrafted myofibers and their closest neighboring myofibers was evaluated with the Fisher exact test. Since the sample size for our BLI data was small, we used bootstrapping to evaluate the results [44]. Data are presented as the mean ± standard deviation (SD). A *p* value of <0.05 was considered to be statistically significant.

## Results

### In vivo bioluminescence imaging reveals acute donor cell loss within the first week following transplantation

**Fig. 1:**
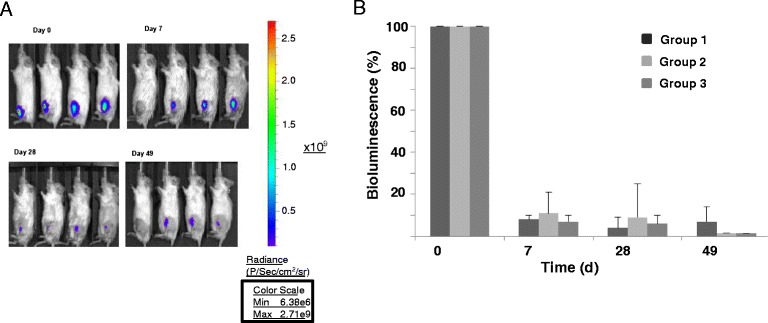
Monitoring of hMPCs after transplantation. **a** Representative bioluminescence images of mice on days 0, 7, 28, and 49 post-transplantation with 2 × 10^6^ hMPCs and a 4–5-week period of iNMES training (*n* = 4). Signal activity was detected in the regions of interest (ROI), encompassing the TA where the cells were originally injected, and quantified as photons/s. **b** Quantitative analysis of bioluminescence as a function of time following transplantation. Within the first week post-transplantation, <10 % of the original signal was detected in each of the three groups analyzed (group 1, *N* = 3; group 2, *N* = 7; group 3, *N* = 6). Less than 5 % of the transplanted hMPCs were retained at the end of the 7-week period following transplantation

We initially used BLI to evaluate the efficiency of engraftment and the survival of engrafted LHCN cells. We acquired luminometry images for most mice on day 0, at about 2 h after injection of the hMPCs, and then at 7 and 28 days after transplantation. We also examined some mice on a weekly basis for up to 7 weeks post-engraftment (Fig. 1a). Injection of 5 × 10^5^ (group 1) or 2 × 10^6^ hMPCs (groups 2 and 3) into the X-irradiated and CTX-intoxicated murine TA compartment resulted in a robust bioluminescence signal at day 0 of injection. We observed a significant decay in the bioluminescent intensity for all three experimental groups, indicating <10 % average retention of hMPCs after 1 week post-transplantation. A gradual reduction in the bioluminescent intensity was observed thereafter, with results at 4 and 7 weeks corresponding to average cell survival rates of 6 and 3 %, respectively (Fig. 1b). No differences were observed in the signals obtained from the grafts between 7 and 28 days post-injection for groups 2 and 3 (two-tailed test; *p* = 0.38 and *p* = 0.34, respectively). Overall, our data indicate that the vast majority of injected hMPCs failed to survive under each of the conditions tested.

### iNMES promotes hMPC engraftment and maturation

We collected the tissues formed by the engrafted LHCN cells at 4 to 5 weeks after transplantation and characterized them. We first compared the mass of the engrafted muscle with that of control mouse TA muscles. The grafts, which included the tissue formed by the injected hMPCs as well as connective tissue and any remaining murine tissue that resisted CTX intoxication, varied from 4 to 14 mg in mass (mean ± SD, 9.9 ± 6.1 for group 1; 9.1 ± 2.7 for group 2, without iNMES; 12.5 ± 11.6 for group 3, with iNMES). By contrast, TA muscles in healthy mice have a mass of 31–51 mg (mean ± SD, 43.3 ± 2.8 mg). Thus, the yield of tissue in the grafts is too variable for any differences to reach statistical significance.

**Fig. 2:**
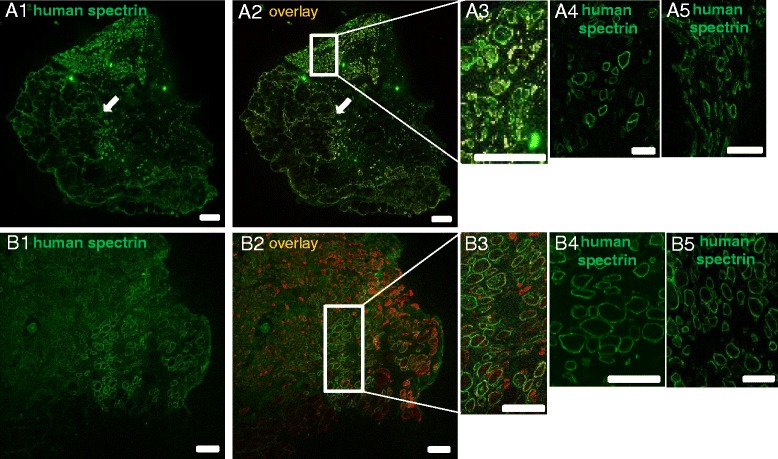
Grafts formed by 2 × 10^6^ hMPCs with and without iNMES. The presence of human muscle fibers in murine hindlimbs was evaluated at 4 weeks post-transplantation. An antibody specific for human β-spectrin was used to stain cross sections of grafts by immunofluorescence; antibodies to desmin were used to stain both murine and human muscle fibers. The results show more human β-spectrin-positive myofibers (*green*), of larger size and in denser packing, in the grafts of mice treated with iNMES (group 3; *B1*, *B4*, *B5*) compared with untreated mice (group 2; *A1*, *A4*, *A5*). *White arrows* in *A1* and *A2* indicate a region of endogenous murine muscle that persisted following X-irradiation and CTX treatments. All cells labeled with antibodies to human β-spectrin in both groups 2 and 3 labeled with antibodies to desmin (*red* in merged images, *A2* and *B2*, respectively), confirming their identity as myofibers. *A3* and *B3* show the *boxed areas* in *A2* and *B2* at higher magnification. *A4* and *A5*, and *B4* and *B5*, show muscle fibers of human regions of two other grafts from mice treated without (*A4*, *A5*) and with (*B4*, *B5*) iNMES. Scale bars = 50 μm

To examine the nature of the tissues in the grafts formed by the LHCN cells, we prepared frozen cross sections and labeled them by immunofluorescence methods with antibodies specific for human β-spectrin, a protein present at the sarcolemma of mature myofibers [45]. As shown in Fig. 2, the anterior tibial compartment of the hindlimb, formerly occupied by the murine TA muscle, contains tissue of human origin, as it stains with the antibody specific for human β-spectrin (green in Fig. 2). These cells also label for desmin (red in Fig. 2), an intermediate filament protein expressed only in muscle [46–49], indicating that the cells positive for β-spectrin at the sarcolemma are indeed muscle fibers of human origin. No fibers in this region fail to stain with the antibody to human β-spectrin, suggesting that all are generated at least in part by the fusion of hMPCs to form myofibers. By contrast, murine myofibers, identified by labeling for desmin but not for human β-spectrin, are apparent in regions of the hindlimb that were not exposed to CTX. Our results indicate that transplantation of hMPCs into murine hindlimb exposed to X-irradiation and CTX can yield muscle tissue of human origin.

**Fig. 3:**
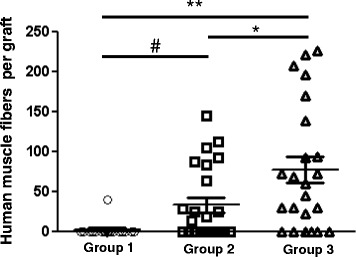
Number of human myofibers per xenograft. The scattergram shows the distribution of the total number of engrafted human muscle fibers in each graft following hMPC transplantation, determined by counting the number of fibers labeled with antibodies to human β-spectrin in cross sections. Only one graft with human tissue, containing 39 human myofibers, was obtained from a total of 18 mice in group 1. Groups 2 and 3 showed more grafts with higher numbers of muscle fibers of human origin, with a range of 13–145 and 22–226 myofibers per cross section sampled, respectively. Differences between groups were significant by *t* tests (#*p* < 0.01; *p < 0.05; **p < 0.001)

Quantitation of the results of grafts formed after injection of 5 × 10^5^ LHCN cells revealed that only one of 18 engrafted limbs (~6 %) developed muscle fibers of human origin. By contrast, approximately 50 and 75 % of limbs injected with 2 × 10^6^ LHCN hMPCs developed muscle fibers of human origin under control conditions and following iNMES, respectively (Fig. 3; Table 1). These differences were significant (*p* < 0.05).

**Table 1:** Properties of xenografts

	Group 1	Group 2	Group 3
Number of hMPCs injected	5 × 10^5^	2 × 10^6^	2 × 10^6^ + iNMES
Mice (number)	18	24	23
Mice with human fibers (%)	6	48	74
Average number of human fibers/graft^a^	2.2 ± 8.9	31.8 ± 45.0	76.9 ± 77.9
Minimum Feret’s diameter (μm)^a^	8.6 ± 3.4	12.4 ± 7.1	13.5 ± 6.3
Average distance to closest fiber (μm)^a^	ND	7.6 ± 5.6	4.2 ± 4.2

*ND* not determined

^a^Mean ± SD

**Fig. 4:**
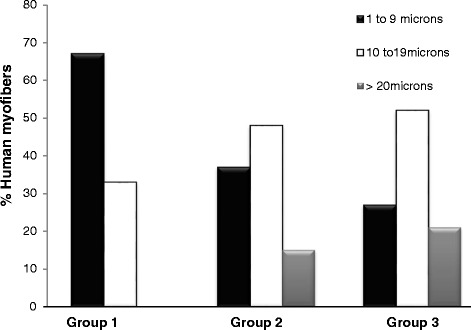
Size distribution of human muscle fibers cells in the xenografts. Minimum Feret’s diameters of the human muscle fibers were measured. Values were binned (1–9, 10–19, and ≥20 μm), and each bin was expressed as percent of the total. Significantly more (21 %) human muscle fiber in the mice of group 3 reached larger sizes (>20 μm) compared with those detected in mice from groups 1 and 2 (0 and 15 %, respectively) (chi-square test, *p* < 0.00001)

We used three additional morphological criteria—number and size of the myofibers, and interfiber spacing—to assess the human muscle tissue in the LHCN xenografts. Quantitation of the number of muscle fibers of human origin in the graft, identified by immunofluorescence labeling with antibodies to human β-spectrin, shows that more large fibers formed following injection of 2 × 10^6^ hMPCs and treatment with iNMES than under the two other conditions we tested. In the one graft that formed from LHCN cells injected at a dose of 5 × 10^5^, 22 of the 33 myofibers present in the graft were <10 μm in diameter, while 33 % reached diameters of 10–19 μm (11/33, Fig. 4). The grafts formed in group 2, injected with 2 × 10^6^ LHCN hMPCs but not exposed to iNMES, contained more muscle fibers of larger size, ranging in number from 13 to 145 per graft (Fig. 3), with nearly half (128 of 267 or 48 %) being >10 μm in diameter (Fig. 4). These results suggest that increasing the number of injected hMPCs from 5 × 10^5^ to 2 × 10^6^ promotes successful engraftment and muscle development (*p* < 0.01).

Injection of 2 × 10^6^ LHCN hMPCs followed by iNMES led to grafts with the highest numbers of human muscle fibers of the largest size (Table 1). Mice in this group developed grafts containing 76.9 ± 77.9 (mean ± SD) human myofibers, compared to 31.8 ± 45.0 in group 2 (*p* < 0.05). The human myofibers in the grafts of group 3 were also larger, with 52 % reaching diameters of 10–19 μm diameter, and 21 % reaching diameters of ≥20 μm (Fig. 4). The largest number of myofibers of human origin in grafts exposed to iNMES was 226, ~10 % of the number of myofibers in a normal mouse TA muscle [50]. At 4–5 weeks after transplantation of LHCN cells, we observed differences in the distribution of myofiber sizes between groups 2 and 3, with grafts in group 3 containing a higher percentage of larger myofibers ≥20 μm in size and a smaller percentage of myofibers of 1–9 μm in size (22 % large, 27 % small) compared with group 2 (15 % large, 37 % small; *p* < 0.00001). Therefore, iNMES not only promotes the success of engraftment but also increases the number and size of the human muscle fibers formed by the engrafted hMPCs.

**Fig. 5:**
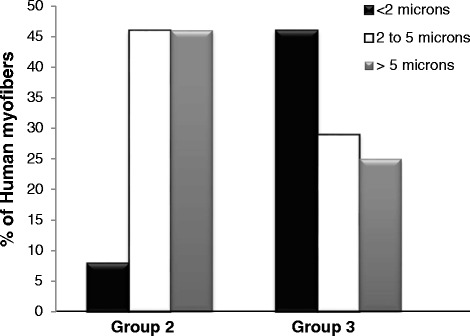
Nearest neighbor distances of the human muscle fibers in the grafts. Distances between the largest muscle fibers of human origin in groups 2 and 3, and their nearest neighbors were measured and binned in three groups (<2, 2–5, and >5 μm). Each bin was expressed as percent of the total. The histograms show the distribution of distances between neighboring human muscle fibers. Human myofibers in the grafts in group 3 were more closely spaced than those in group 2 (Fisher’s exact test, *p* < 0.0001)

We further evaluated the integrity of the human muscle tissue in the grafts formed by the LHCN hMPCs by measuring the distances from the largest myofibers of human origin in groups 2 and 3 to their nearest neighbors. Our results show that the largest myofibers in group 3 are closer to their nearest neighbors (4.2 ± 4.2 μm) than those in group 2 (7.6 ± 5.6 μm; *p* < 0.0001), consistent with the larger number of myofibers in these grafts and their closer packing (Fig. 5; Table 1).

**Fig. 6:**
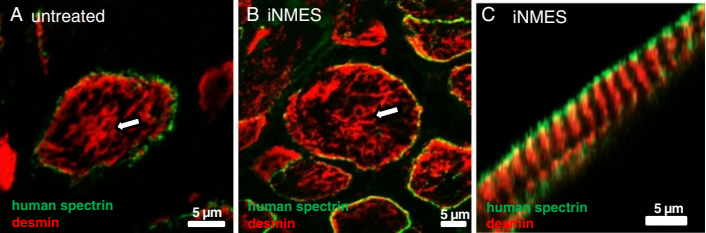
Reticulum of desmin in muscle fibers of human origin. Unfixed cross sections of the xenografts of groups 2 and 3 were labeled with antibodies to desmin (**a**, **b**). The reticular pattern of staining for desmin (*red*) in the myoplasm of human myofibers (*white arrowheads*), identified by the presence of human β-spectrin (*green*) at the sarcolemma, indicates that the larger myofibers in both groups 2 (**a**) and 3 (**b**) are fully differentiated. **c** A fiber from group 3 with desmin in striations, indicating that the reticulum of desmin is fully organized around myofibrils. Scale bars = 5 μm

We addressed the state of differentiation of the human muscle fibers in the grafts formed by LHCN cells in groups 2 and 3 by examining cross sections of the engrafted tissues labeled with antibodies to desmin. Our results indicate that nearly all the larger myofibers of human origin in groups 2 and 3 have a reticulum of desmin in the myoplasm (Fig. 6a, b), formed as desmin aligns with myofibrils as they striate (Fig. 6c). Desmin normally assembles into a filamentous reticulum around the Z-disks at the later stages of assembly of the contractile apparatus, as the process of differentiation into myofibers is completed [46, 48, 51]. The presence of a striated reticulum of desmin in the larger human muscle fibers in the grafts therefore suggests that these cells have undergone terminal differentiation.

Nuclear labeling with DAPI provided an additional method to determine if engrafted muscle fibers of human origin were terminally differentiated. In developing or regenerating muscle tissue, nuclei are frequently found in the middle of the cell, whereas in healthy, fully differentiated human muscle tissue (but not in regenerated mouse muscle), nearly all of the myonuclei are located peripherally, immediately adjacent to the sarcolemma. Evaluation of human myofibers in the grafts formed by LHCN cells, co-labeled with antibodies specific for human lamin A/C, showed that only 17.5 (36/205) and 13.7 % (69/505) had central nuclei of human origin in groups 2 and 3, respectively. (Murine myonuclei comprised <1.5 % of all central myonuclei in fibers positive for human β-spectrin and so did not contribute significantly to the population of central nuclei: see below). Thus, grafts in both groups 2 and 3 likely consisted of a mixed population of fully and partially differentiated myofibers of human origin, with >80 % being fully differentiated. Overall, our results suggest that most of the grafts formed by engrafted LHCN hMPCs differentiate into mature human muscle fibers during the 4 to 5-week period post-transplantation, and that, although the formation, growth, and maturation of the grafts are promoted by iNMES, iNMES is not required for differentiation.

**Fig. 7:**
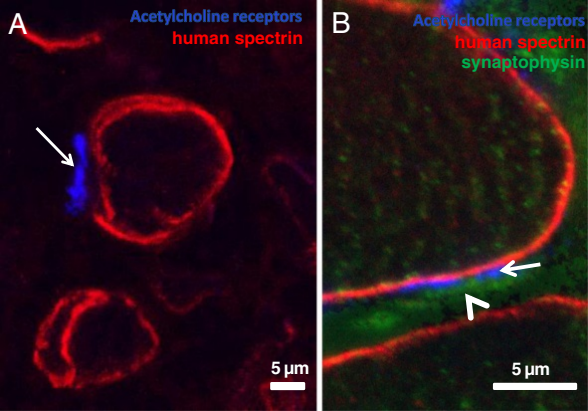
Neuromuscular junctions in the xenografts. Cross sections of unfixed xenografts from group 3 were labeled with Alexa Fluor 633-α-bungarotoxin to visualize postsynaptic acetylcholine receptors (AChRs) (*blue*; **a**, **b**, *arrows*) and with antibodies to human β-spectrin (*red*; **a**, **b**) and to synaptophysin (*green*; **b**, *arrowhead*) to visualize the sarcolemma of human myofibers and presynaptic structures, respectively. The results show the accumulation of AChRs at the sarcolemma of human myofibers (**a**, **b**; *arrows*) that correspond with sites of innervation (**b**; *arrowhead*). Scale bars = 5 μm

The presence of large, differentiated myofibers in the grafts suggests that the myofibers formed by the LHCN hMPCS are innervated. In the absence of innervation, these fibers would atrophy significantly and would consequently be reduced in size [52]. To test for innervation, we labeled the grafts of group 3 with a fluorescent derivative of α-bungarotoxin to detect the large clusters of acetylcholine receptors (AChRs) in the postsynaptic membrane of the neuromuscular junction. We found clustered AChRs, suggesting that innervation had occurred within 4 weeks after transplantation of hMPCs (Fig. 7). As the AChRs of non-innervated muscle cells can also aggregate [53, 54], we also immunolabeled sections with antibodies to synaptophysin, a protein localized presynaptically at motor nerve terminals [55, 56]. Immunostaining for synaptophysin was faint but present adjacent to the clusters of AChRs on the surface of the human myofibers in the graft (Fig. 7b), indicative of an intact neuromuscular junction. We conclude that human myofibers in the grafts formed by LHCN cells are indeed innervated.

**Fig. 8:**
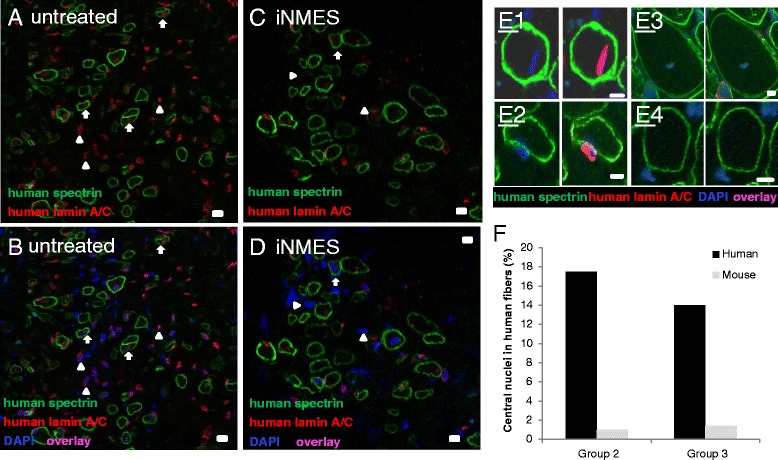
Centrally located myonuclei of human and murine origin in xenografts. Unfixed cross sections were immunolabeled with antibodies to human β-spectrin (*green*) and human lamin A/C (*red*) and counterstained with DAPI (*blue*) to label all nuclei. Human myonuclei, positive for DAPI and human lamin A/C, and murine myonuclei, positive for DAPI but not for human lamin A/C, were quantitated in human myofibers and identified by the presence of β-spectrin at the sarcolemma. Images of human myofibers in group 2 (**a**, **b**) and group 3 (**c**, **d**) show labeling for spectrin and lamin A/C (**a**, **c**) and for both proteins and DAPI (**b**, **d**). **a**, **b** *Arrows* point to myofibers with nuclei labeled with antibodies to human lamin A/C. *Arrowheads* point to nuclei labeled with antibodies to human lamin A/C lying outside of muscle fibers. **c**, **d** *Arrow* points to a human myofiber with a nucleus that is unlabeled by antibodies to human lamin A/C. *Arrowheads* point to nuclei that lie outside of muscle fibers and that do not label with antibodies to human lamin A/C. **e** High magnification examples of human fibers with a myonucleus that labels with anti-human lamin A/C (*E1*), a nucleus lying outside a human fiber that labels with anti-human lamin A/C (*E2*), a human fiber with a myonucleus that fails to label for human lamin A/C (*E3*), and a nucleus that fails to label for human lamin A/C lying outside a human fiber (*E4*). **f** Percent of centrally located human and murine myonuclei detected in human muscle fibers in groups 2 and 3 at 4–5-weeks post-transplantation. Of the human fibers examined, <20 % was centrally nucleated (group 2 = 36/205, or 17.5 %; group 3 = 69/505 or 13.7 %). Only 2 myonuclei out of 205 (1 %) in group 2 and 7 myonuclei out of 505 (1.4 %) in group 3 failed to label with antibodies to human lamin A/C. Scale bars = 10 μm

We next considered the possibility that a small number of endogenous murine satellite cells might have resisted X-irradiation and could therefore have expanded and fused with the transplanted hMPCs following CTX intoxification, leading to the formation of hybrid murine-human myofibers. In the absence of a mouse-specific lamin A/C antibody, we identified murine myonuclei as those DAPI-stained myonuclei within myofibers that labeled positively for human β-spectrin but that failed to label with antibodies specific for human lamin A/C [6–8, 38, 41–43]. Images of sections of a graft labeled with antibodies specific for human β-spectrin and human lamin A/C, and counterstained with DAPI to visualize nuclei, are shown at both low and high magnification in Fig. 8. Quantitation of central nuclei of murine origin in human myofibers showed that their numbers were extremely low both in group 2 (2/205, or 1 %) and group 3 (7/505, or 1.4 %). Nearly all myonuclei are centrally located in murine muscles at 4 weeks after CTX intoxication, however. Thus, our results indicate that there was minimal murine contamination of the human myofibers formed by LHCN hMPCS.

Consistent with these results, qualitative assessment of the human muscle grafts formed by LHCN hMPCs across the three experimental groups revealed that the vast majority of the murine myofibers were ablated following X-irradiation and CTX treatment. Those remaining were typically at the edge of the graft containing human muscle tissue, indicating that the human muscle and murine muscle tended to segregate from one another spatially. Therefore, the combination of X-irradiation and CTX treatments of the murine TA muscle not only eliminated all or nearly all of the tissue of murine origin but also inhibited the formation of hybrid human-murine myofibers.

**Fig. 9:**
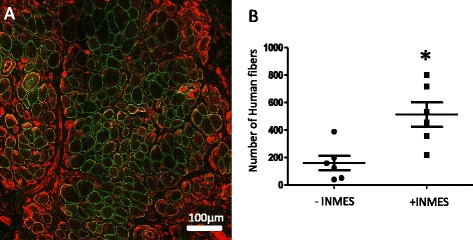
Xenografts generated with FSHD hMPCs. Xenografts were generated as for groups 2 and 3 (see Fig. 2), using FSHD hMPCs. **a** A graft labeled with antibodies to desmin (*red*) and to human β-spectrin (*green*) shows many large muscle fibers of human origin that are up to 53 μm in diameter. **b** Scattergram of counts of human muscle fibers in six xenografts treated with iNMES and six xenografts that remained untreated. iNMES significantly increases the number of human fibers in the grafts formed by FSHD hMPCs (*p < 0.01)

Finally, we performed preliminary studies to determine if the methods we had developed for LHCN cells could be applied to an immortalized human myogenic cell line derived from an individual with FSHD. As shown in Fig. 9a, engraftment of FSHD hMPCs leads to the formation of muscle tissue that has large fibers of human origin. Figure 9b shows that exposure of the grafts to iNMES also significantly increases the sizes of the grafts formed by FSHD hMPCs (*p* < 0.01). Thus, our methods can indeed be applied to other immortalized lines of hMPCS, including those from individuals with muscular dystrophies.

## Discussion

Previous studies of grafts of hMPCs into immunodeficient or dystrophic murine muscle have shown that the regenerative potential of the endogenous muscle cell population typically is only partially eliminated, leading to the formation of hybrid human-murine myofibers and hence the expression of both human and murine muscle proteins. The purpose of our study was to discover if suppressing the myogenic potential in the hindlimb of the host mouse would permit muscle fibers to form that are largely, if not exclusively, human in origin. Our ultimate goal was to develop mature skeletal muscle tissue in the murine hindlimb consisting exclusively of human myofibers, with few or no murine myonuclei. We used a dose of X-rays sufficient to block myogenesis in immunodeficient NOD-Rag mice (25 Gy), followed by injection of the myotoxin CTX to eliminate their TA muscles. We then injected the TA compartment with hMPCs expressing luciferase (LHCN cells [21]). Our results indicate that hMPCs do indeed generate mature innervated, human myofibers in mice treated in this way. They also show that contamination of the human fibers with murine myonuclei is minimal (<1.5 %) and that iNMES promotes the survival and development of human myofibers formed by the engrafted hMPCs, including those derived from dystrophic muscle. To our knowledge, ours are the first results to demonstrate that human myofibers can be generated in mice without the incorporation of significant numbers of murine myonuclei. They further establish that our methods can generate human muscle tissue in mice from hMPCs from an individual with FSHD.

Although our results are unique, our methods are based on those used by a number of other laboratories. Several different immunodeficient mouse strains have been used for xenografting, including nude, SCID, and NOD-Rag. We have studied the latter, which have also been examined by others [6–8, 10, 39, 40]. The death of muscle fibers prior to studies of regeneration or engraftment can be induced by cryodamage [6–8, 10, 39–41], but anesthetics, such as bupivacaine, Ba^2+^, or toxins such as notexin and cardiotoxin, have been used more widely for this purpose and in some cases have been directly compared [9, 57–60]. In our current experiments, we chose to use cardiotoxin, with which we were already familiar [33]. As in our previous studies of muscle injury and regeneration [34], see also [61], we used 25 Gy irradiation to prevent murine satellite cells from regenerating, rather than 18–20 Gy irradiation favored by others [9, 10, 57, 60, 62]. To identify human myonuclei and fibers, we used antibodies specific for human lamin A/C and β-spectrin, as described [6–8, 10, 38–41]. Our results show that this unique combination of reagents and methods leads to engraftment of hMPCs and the formation of fibers that are almost exclusively human in origin.

Our approach also involves a novel use of iNMES to promote the formation of human myofibers in the mouse hindlimb. iNMES can promote both the engraftment of myogenic cells and the recovery of muscle from injury [25, 27–30, 63], but the mechanisms by which it acts are still poorly understood. iNMES may modulate the environment in the hindlimb, for example, by suppressing inflammation, reducing proliferation of murine fibroblasts, increasing angiogenesis, or promoting the innervation of the engrafted tissue by murine motor neurons. In clinical settings, electrotherapy has the potential to produce a long-lasting reduction in inflammation [64], though we are not aware of quantitative studies of the effects of iNMES on inflammation of muscle. The literature is also mute regarding the effects of electrical stimulation on the development of fibrosis in developing, damaged, or regenerating muscle. Electrical stimulation has, however, been reported to increase angiogenesis in muscle [25, 26] and regeneration of motor neurons [65, 66], perhaps by increasing the levels of trophic factors such as vascular endothelial growth factor (VEGF) and neurotrophins [67, 68]. Furthermore, iNMES can promote the proliferation and differentiation of both myoblasts and myotubes in vitro [22, 24, 69, 70]. Its application in our in vivo studies may therefore increase the number of hMPCs that survive following injection, as well as their state of maturity at the time we assay the grafts.

Despite the remarkable effects of iNMES, the grafts that form with our procedures remain small. The largest grafts formed by either LHCN or FSHD hMPCs contain only ~10–30 % of the number of fibers of a mature TA muscle in the mouse, and only a subset of these have diameters similar to mature murine TA fibers. One possible explanation of the small sizes of the xenografts is the massive loss of hMPCs within the first week following injection into the hindlimb, as indicated by our bioluminescence imaging studies (Fig. 1), which occurs with LHCN hMPCs at either dose of cells injected, with or without iNMES. Although our luminometric data illustrating this acute cell loss show some variability, it is clear that, regardless of treatment, >90 % of the bioluminescence signal emitted by the luciferase-expressing hMPCs is lost within the first week in vivo.

Cell death caused by the failure of the myogenic cells to attach to matrix or other physical structures in the limb, known as anoikis [70], is likely to be a major contributor to this loss. Consistent with this, co-injection of human myoblasts with extracellular matrix components can improve their survival, proliferation, and migration, leading to a more successful engraftment [71, 72]. Moreover, introduction of a fibrin gel, which could provide additional adhesion sites, has been reported to promote myoblast transplantation in mice [73]. Organized three-dimensional matrices, which have been studied in vitro [68, 69, 74–76], may also be useful in providing adhesive surfaces and thereby promoting survival and differentiation, to increase the size of the grafts that we can generate in vivo with the use of iNMES.

The proliferative ability of the hMPCs that survive engraftment may be limited, despite the benefits of iNMES. Growth factors (IGF-1; [75, 77, 78]) or modulatory factors such as the binding site of the ActRIIB receptor, which binds myostatin and other members of the TGF-β family of polypeptide hormones [18], have been reported to improve engraftment. Although they were tested under different conditions than those used here, they may also promote the growth and differentiation of the grafts formed by hMPCs.

Yet another possibility is that the LHCN cells used in our studies have only a limited ability to adhere, proliferate, and differentiate in vivo, perhaps because they have been maintained in culture through many (>100) population doublings. In this case, other immortalized lines of hMPCs may engraft and form human muscle tissue in mice more efficiently. Our initial observations on the efficiency of engraftment of FSHD cells (Fig. 9), which have been through <30 passages and which generate xenografts with approximately fivefold more muscle fibers, is consistent with this. It is also conceivable that the grafts were still growing at the time we harvested them and that maintaining them for longer periods of time in the mice will yield grafts of human muscle that are significantly larger and better organized. We have been unable to identify Pax7-positive cells in the satellite cell niche in our LHCN grafts, however, suggesting that further growth may be slow, if it occurs at all. This agrees with results of Mamchaoui et al. [39] but not with those of several other laboratories [6, 7, 9, 40, 57, 79]. We are currently pursuing experiments to address these questions, with the aim of increasing the size and improving the morphology and physiology of the muscles generated by our methods.

## Conclusions

Although our research is still at an early stage, it has nevertheless succeeded in generating xenografts of human muscle tissue in mice that are unique. These grafts are formed from muscle precursor cells in a compartment that is essentially devoid of murine muscle, in contrast to grafts of bundles of intact muscle fibers sewn into pre-existing murine muscle, as recently described [7]. Furthermore, the grafts consist of myofibers that are fully differentiated and innervated by motor neurons. Although we have not yet studied the muscles formed by the grafts in isolation, our preliminary observations indicate that they do indeed contract in response to electrical stimulation. Perhaps most significantly, unlike other studies of transplantation of human myoblasts into mice, which generate hybrid fibers with both human and murine myonuclei, the myofibers in the grafts we have produced are essentially free of murine myonuclei and are therefore largely human in origin. This indicates that the methods we have used to eliminate the TA muscle of the mouse and to prevent it from regenerating were both successful and compatible with the formation of human myofibers from injected hMPCs. Our results further suggest that the methods we have developed will also be useful for generating human muscle tissue in mice from individuals with FSHD. We look forward to using our methods not only to provide novel tools to study the potentially unique properties of human skeletal muscle but also to develop new xenograft models for human myopathies and muscular dystrophies.

## Abbreviations

AChRs: acetylcholine receptors
BLI: bioluminescence imaging
CTX: cardiotoxin
DAPI: 4′,6-diamidine-2′-phenylindole dihydrochloride
hMPCs: human myogenic precursor cells
iNMES: intermittent neuromuscular electrical stimulation
LHCN: lox-hTERT hygromycin + cdk4-neo; immortalized human myogenic cell line
MOM: mouse-on-mouse
NOD-Rag: NOD-Rag1^null^IL2rγ^null^
PBS: phosphate-buffered saline
TA: tibialis anterior
TBS: Tris-buffered saline
